# Genetic relationship and diversity among coconut (*Cocos nucifera* L.) accessions revealed through SCoT analysis

**DOI:** 10.1007/s13205-015-0304-7

**Published:** 2015-05-07

**Authors:** M. K. Rajesh, A. A. Sabana, K. E. Rachana, Shafeeq Rahman, B. A. Jerard, Anitha Karun

**Affiliations:** Division of Crop Improvement, ICAR-Central Plantation Crops Research Institute, Kasaragod, 671124 Kerala India

**Keywords:** *Cocos nucifera*, Genetic diversity, Gene-targeted markers, SCoT

## Abstract

Coconut (*Cocos nucifera* L.) is one of the important palms grown both as a homestead and plantation crop in countries and most island territories of tropical regions. Different DNA-based marker systems have been utilized to assess the extent of genetic diversity in coconut. Advances in genomics research have resulted in the development of novel gene-targeted markers. In the present study, we have used a simple and novel marker system, start codon targeted polymorphism (SCoT), for its evaluation as a potential marker system in coconut. SCoT markers were utilized for assessment of genetic diversity in 23 coconut accessions (10 talls and 13 dwarfs), representing different geographical regions. Out of 25 SCoT primers screened, 15 primers were selected for this study based on their consistent amplification patterns. A total of 102 scorable bands were produced by the 15 primers, 88 % of which were polymorphic. The scored data were used to construct a similarity matrix. The similarity coefficient values ranged between 0.37 and 0.91. These coefficients were utilized to construct a dendrogram using the unweighted pair group of arithmetic means (UPGMA). The extent of genetic diversity observed based on SCoT analysis of coconut accessions was comparable to earlier findings using other marker systems. Tall and dwarf coconut accessions were clearly demarcated, and in general, coconut accessions from the same geographical region clustered together. The results indicate the potential of SCoT markers to be utilized as molecular markers to detect DNA polymorphism in coconut accessions.

## Introduction

Coconut (*Cocos nucifera L.*), belonging to the family Arecaceae, is the only reported species under the genus *Cocos*. In the major growing countries in Asia, it is grown as both as a homestead as well as a plantation crop over large areas. Coconut palms are found abundantly in coastal regions of most tropical islands, significantly contributing to the sustenance of fragile island ecosystems and the livelihood of people. Considering the versatile role of coconut palms in providing food, nutrition, fibers, beverage, medicine, shelter, and wide range of handicrafts (from different parts of the palm) throughout its lifetime, the coconut palm is termed as ‘*Kalpavriksha*’ meaning ‘tree of heaven’ or ‘tree of life’.

Tall (‘*typica*’) and dwarf (‘*nana*’) are two main categories of coconut palms. The Talls are naturally cross-pollinating types, have more economic value, are vigorous growing, comparatively late to flowering and the fruits are with intermediate colors of brown, green, yellow, orange among individual palms. Dwarfs, in contrast, are naturally self-pollinating types with reduced growth habitat, early flowering and produce large number of medium to small, distinctly colored (green or yellow or orange or brown) fruits (Dasanayaka et al. 2009). Many efforts are ongoing in coconut-growing countries to conserve the rich natural diversity existing in coconut germplasm collections for further utilization in crop improvement programs so that it becomes a more profitable crop for small-farm holders, who constitute the vast majority of coconut growers (Batugal et al. 2005). As a first step towards this goal, assessment of genetic diversity assumes significance for germplasm conservation and their subsequent utilization.

The repertoire of markers utilized for characterizing genetic diversity in coconut can be categorized into morphological, biochemical and DNA-based. Morphological traits like seed germination time (Bourdeix et al. 1993), fruit component analysis (Harries 1978), floral biology and pollination behavior (Sangare et al. 1978; Ratnambal et al. 2003), foliar traits (N’Cho et al. 1993; Arunachalam et al. 2005) and biochemical parameters like foliar polyphenols (Jay et al. 1989), proteins (Cardena et al. 1998) and isozymes (Benoit and Ghesquiere 1984) have formed the basis of many studies in coconut diversity analysis. However, morphological and biochemical markers possess many drawbacks like being limited in number, show modest levels of polymorphism and low heritability and can be influenced by developmental stages of the plant and varied environmental factors. DNA-based markers can overcome the limitations of the above marker systems. DNA-based markers used in coconut include RAPD (Ashburner et al. 1997; Upadhyay et al. 2004; Ritto et al. 2008), RFLP (Lebrun et al. 1998), AFLP (Perera et al. 1998; Teulat et al. 2000), ISTR (Rohde et al. 1995), ISSR (Manimekalai and Nagarajan 2006a) and SSRs (Perera et al. 1999; Rivera et al. 1999; Merrow et al. 2003; Rajesh et al. 2008a, b). These markers have been applied for assessment of genetic diversity within coconut germplasm, construction of linkage maps for mapping genes or quantitative trait loci (QTL) controlling agronomically important traits (Baudouin et al. 2006; Herran et al. 2000) and trait identification (Rajesh et al. 2013).

Recent advances in genomic research has resulted in a change of preference from the use of random DNA markers to gene-targeted, functional markers and the development of novel DNA-based marker systems (Gupta and Rustgi 2004; Poczai et al. 2013). The development of functional markers has been simplified by an explosion of resources in public genomic databases (Andersen and Lubberstedt 2003). Molecular markers developed from the transcribed region of the genome have the ability to reveal polymorphism, which might be directly related to gene function (Poczai et al. 2013). Start codon targeted polymorphism (SCoT) is a simple and novel marker system first described by Collard and Mackill (2009), which is based on the short conserved region flanking the ATG translation start codon in plant genes. The technique uses single primers designed to anneal to the flanking regions of the ATG initiation codon on both DNA strands. SCoT polymorphism marker technique has been successfully applied in rice (Collard and Mackill 2009), peanut (Xiong et al. 2010), longan (Chen et al. 2010), mango (Luo et al. 2010, 2011), citrus (Han et al. 2011), grapes (Zhang et al. 2011), potato (Gorji et al. 2011), persimmon (Deng et al. 2012), orchids (Bhattacharyya et al. 2013), *Jatropa* (Mulpuri et al. 2013), tritordeum (Cabo et al. 2014) and sugarcane (Que et al. 2014). In the current study, we have used SCoT markers to assess the extent of genetic diversity in a worldwide collection of coconut germplasm accessions with an aim of evaluating the efficiency of the marker system in coconut. This is the first report on the use of SCoT markers in analyzing coconut germplasm for genetic diversity and phylogenetic relationships.

## Materials and methods

### Plant material and DNA extraction

A total of 23 coconut accessions, comprising 10 tall and 13 dwarf accessions, representing different geographical regions, were used for this study (Table 1). Fresh spindle leaves were collected from four palms per accession, which are conserved at field gene banks of Central Plantation Crops Research Institute of the Indian Council of Agricultural Research (ICAR)—a National Active Germplasm Site for coconut. DNA was extracted from the total of 96 samples using a modified SDS method as described by Rajesh et al. (2013). The quantity and quality of extracted DNA were verified using the spectrophotometer and agarose gel electrophoresis. DNA from four palms of each accession was pooled together thus making a total of 23 DNA samples. Extracted DNA was stored at −20 °C till further use.

**Table 1:** List of coconut accessions used for the present study

Sl. No.	Accession	Abbreviation	Geographic origin
Tall accessions			
1	West Coast tall	WCT	India
2	Cochin China tall	CCNT	Vietnam
3	Philippines ordinary tall	PHOT	The Philippines
4	Laccadive ordinary tall	LCT	Lakshadweep Islands, India
5	Borneo tall	BONT	Indonesia
6	Laccadive mini micro tall	LMMT	Lakshadweep Islands, India
7	Klapawangi tall	KWGT	Malaysia
8	Andaman ordinary tall	ADOT	Andaman Islands, India
9	Andaman giant tall	ADGT	Andaman islands, India
10	San Ramon Tall	SNRT	The Philippines
Dwarf accessions			
11	Chowghat green dwarf	CGD	India
12	Chowghat orange dwarf	COD	India
13	Malayan yellow dwarf	MYD	Malaysia
14	Malayan orange dwarf	MOD	Malaysia
15	Sri Lankan red dwarf	SLRD	Sri Lanka
16	Sri Lankan green dwarf	SLGD	Sri Lanka
17	Kenthali orange dwarf	KTOD	India
18	Gudanjali green dwarf	GDGD	India
19	Nikkore orange dwarf	NKOD	Papua New Guinea
20	Cameroon red dwarf	CRD	Cameroon
21	Niu Leka green dwarf	NLAD	Fiji
22	Hari Papua orange dwarf	HPOD	French Polynesia
23	Gangabondam green dwarf	GBGD	India

### SCoT marker analysis

All SCoT primers were synthesized from SIGMA (India). Twenty-five SCoT primers (SCoT 1–SCoT 25) described by Collard and Mackill (2009) were initially screened for polymorphism and reproducibility in a subset of six accessions, three each of talls and dwarfs. PCR amplification of these primers was carried out in a thermal cycler in 20 µl volume, with the reaction mixture containing 2 µl of 10× PCR buffer, 0.4 µl dNTPs (10 mM), 1.6 µl primer (10 µM), 0.3 µl *Taq* DNA polymerase (3U/µl), and 3 µl template DNA (10 ng/µl) and 12.7 µl distilled water. Cycling conditions in a thermal cycler (DNA engine: BIORAD) were: initial denaturation at 94 °C for 3 min followed by 34 cycles of 94 °C for 1 min, 52 °C for 1 min, 72 °C for 2 min and final extension at 72 °C for 5 min. All primers were amplified using the same procedure. After amplification, PCR products were mixed with 3 µl of 6× gel loading dye (0.25 % bromophenol blue, 0.25 % xylene cyanol FF, 30 % glycerol in water) and separated on 1.5 % agarose gel in 1× TBE buffer by electrophoresis and stained with ethidium bromide (0.5 µg/ml). A 1 Kb ladder (MBI Fermentas) was used as a molecular size standard. Gels were visualized in a gel documentation system (Bio-Rad). All experiments were repeated twice.

### Data analysis

Only clear and reproducible PCR amplified products from SCoT primer were used for further analysis. The bands were scored as absent (0) or present (1). Software package NTSYS-pc version 2.0 (Rohlf 1993) was used for the further analysis using the scoring results. Genetic similarity analysis between tall and dwarf accessions of coconut was estimated using similarity matrix, generated by calculating Jaccard’s similarity coefficient. These similarity coefficients were then used for cluster analysis and a dendrogram was constructed by the unweighted pair-group method (UPGMA) (Sneath and Sokal 1973). WINBOOT software (Yap and Nelson 1996) was used for the assessment of robustness of the dendrogram typology and the estimation of robustness of cluster analysis.

The average polymorphism information content (PIC) was calculated by applying the formula given by Powell et al. (1996): ${\text{PIC}} = 1 - {{\Sigma }}f_{i}^{ 2}$, where *i* = 1 − *n* where *f*_*i*_ is the frequency of the *i*th allele. The number of alleles refers to the number of scored bands. The frequency of an allele was obtained by dividing the number of accessions where it was found by the total number of accessions. The PIC value provides an estimate of the discriminating power of a marker.

## Results

### Polymorphism detected using SCoT markers

Out of 25 primers tested, 15 primers were chosen for further studies based on clarity of the banding patterns (Table 2). The number of bands generated among the 23 coconut accessions using these 15 primers was 102, which ranged from 3 (SCoT 17) to 11 (SCoT 14), with an average of 6.8 bands/primer. Out of these fragments scored, 89 (87.2 %) were polymorphic. Seven of the primers gave 100 % polymorphism, indicating the capability of SCoT primers to detect high levels of polymorphism among coconut accessions (Table 2). The PIC value ranged from 0.16 (SCoT 4) to 0.47 (SCoT 17).

**Table 2:** Sequence of SCoT primers, the number of scorable polymorphic bands and polymorphism information content (PIC) of each primer

Sl. No.	Primer name	Primer sequence	No. of amplified bands	No. of polymorphic bands	Polymorphism %	PIC value
1	SCOT 1	CAACAATGGCTACCACCA	8	7	87.5	0.36
2	SCOT 4	CAACAATGGCTACCACCT	8	6	75	0.16
3	SCOT 5	CAACAATGGCTACCACGA	7	6	85.71	0.25
4	SCOT 13	ACGACATGGCGACCATCG	9	6	66.67	0.38
5	SCOT 14	ACGACATGGCGACCACGC	11	11	100	0.26
6	SCOT 15	ACGACATGGCGACCGCGA	6	6	100	0.28
7	SCOT 16	ACCATGGCTACCACCGAC	6	3	50	0.38
8	SCOT 17	ACCATGGCTACCACCGAG	3	3	100	0.47
9	SCOT 18	ACCATGGCTACCACCGCC	6	5	83.33	0.26
10	SCOT 19	ACCATGGCTACCACCGGC	6	6	100	0.42
11	SCOT 20	ACCATGGCTACCACCGCG	5	5	100	0.33
12	SCOT 21	ACGACATGGCGACCCACA	9	8	88.89	0.36
13	SCOT 22	AACCATGGCTACCACCAC	7	7	100	0.27
14	SCOT 23	CACCATGGCTACCACCAG	6	5	83.33	0.45
15	SCOT 25	ACCATGGCTACCACCGGG	5	5	100	0.31
		Average	6.8	5.93	87.20	0.33
		Total	102	89		

### Genetic diversity analysis among the coconut accessions

**Fig. 1:**
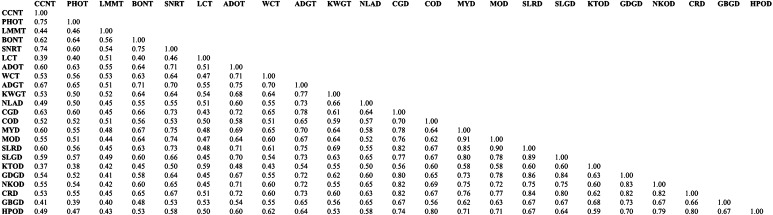
Similarity matrix among 23 coconut accessions based on SCoT markers based on Jaccard’s coefficient

The scored bands were used to calculate the genetic diversity among the 23 coconut accessions. The genetic similarity coefficient between the pair samples was evaluated by calculating the Jaccard’s similarity coefficient based on the proportion of shared bands. The pairwise similarity coefficient was lowest (0.37) between CCNT (a tall accession that originated from Vietnam) and KTOD (a dwarf accession from India). The maximum genetic similarity (0.91) was observed between the two dwarf accessions originating from Malaysia, viz. MYD and MOD (Fig. 1).

### Cluster analysis

**Fig. 2:**
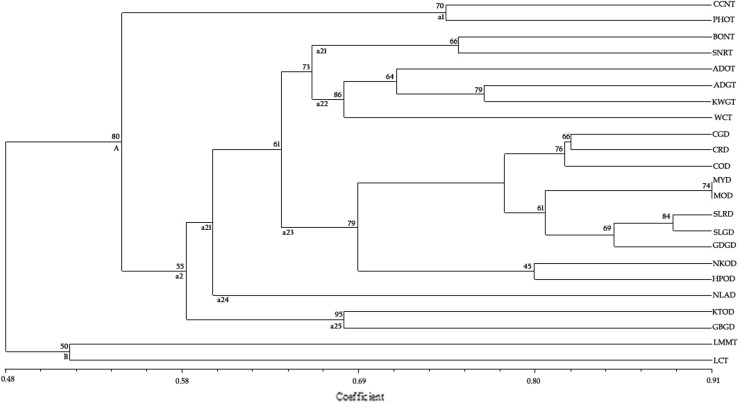
Dendrogram generated from genetic similarity, using Jaccard’s coefficient and UPGMA clustering method of 23 coconut accessions based on SCoT analysis. Numbers on the branches indicate bootstrap support out of 1000 replications

A dendrogram constructed using corresponding genetic similarity coefficients obtained from UPGMA analysis was used to determine the clustering pattern among the coconut accessions analysis (Fig. 2). Two main clusters (designated ‘A’ and ‘B’) were formed diverging at the similarity coefficient of 0.48. The cluster (‘A’) was a large one comprising 21 accessions. Within this cluster (A), there were two sub-clusters (designated ‘a1’ and ‘a2’). Sub-cluster ‘a1’ had a single cluster—comprising two tall accessions that originated from Southeast Asia, viz. CCNT (Vietnam) and PHOT (The Philippines). The second cluster within sub-cluster ‘a2’ had further five clusters (designated ‘a21’ to ‘a25’), which showed a clear separation of tall and dwarf accessions. The first sub-cluster ‘a21’ comprised two Southeast Asian tall accessions that originated, viz. Borneo tall (Indonesia) and San Ramon tall (The Philippines). Cluster ‘a22’ had grouping of four accessions, viz. ADGT and ADOT (from Andaman Islands, India), KWGT (from Malaysia) and West Coast tall (India). All the dwarf accessions formed three clusters; a23, a24 and a25. Cluster ‘a23’ comprised three dwarfs CGD and COD (from India) and CRD (from Africa) were placed within the first sub-cluster, the Malaysian (MOD and MYD) and Sri Lankan Dwarfs (SLRD and SLGD) were placed in the second sub-cluster along with GDGD (from India) and the two Pacific Ocean dwarfs (NKOD and HPOD) were placed in the third sub-cluster. Cluster ‘a24’ had a unique accession, NLAD (from Fiji) and cluster ‘a25’ had two Indian dwarfs, viz. KTOD and GBGD. The second main cluster (‘B’), a distinct one, had two tall accessions from Lakshadweep Islands, India, viz. LCT and LMMT. The clades were supported by reliable bootstrap values (Fig. 2).

## Discussion

A thorough knowledge on genetic relationships among coconut accessions is needed for adopting effective conservation strategies, germplasm utilization and management of the collections. Characterization of coconut germplasm accessions by morphological descriptors or biochemical markers alone have inherent limitations in providing a precise picture of genetic variation. A molecular marker-based assessment across coconut populations in different geographical locations, prior to collecting germplasm for conservation, would form a well-organized strategy for identifying unique populations with specific relevance for utilization in breeding programmes. DNA marker-based assessment of genetic diversity has been utilized by many researchers to assess the extent of genetic diversity among coconut germplasm accessions. A majority of the marker systems utilized, viz. RAPD, DAF, ISSR and AFLP are random, arbitrarily amplified DNA markers. The major advantage of these systems of markers is that there is no need for prior genome sequence of the organism under study. These markers, mostly dominant ones, are generated randomly over the whole genome (multi-loci) and the techniques are non-expensive, technically simple and are capable of generating a relatively large number of markers per sample (Poczai et al. 2013). In spite of these advantages, these techniques are also characterized by a host of weaknesses (Simmons et al. 2007). Since their development, microsatellite markers have been the markers of choice for coconut genetic diversity studies for quite some time. Even though microsatellite markers have numerous advantages, a good separation of alleles is still difficult to achieve using polyacrylamide gels when compared to automated capillary electrophoresis systems. Various other factors also influence the choice of marker system being used, some of them being the availability of technical expertise, equipment and funding (Collard and Mackill 2009).

With the advancements in the field of genomics, novel, gene-targeted functional markers, which possess gene or promoter elements, are gaining prominence and these markers have been reported to offer increased resolution and reproducibility (Poczai et al. 2013). These functional markers, derived from polymorphic sequences, might possibly to be involved in phenotypic trait variation (Andersen and Lubberstedt 2003). One such marker is SCoT, which was based on the observation that short conserved regions of plant genes are bordered by ATG translation start codon (Sawant et al. 1999). Based on this observation, single primers were designed which could anneal to the flanking regions of the ATG initiation codon existing on both DNA strands. The technique is similar to RAPD or ISSR in that a single primer acts as the forward and the reverse primer, amplicons can be visualized by standard agarose gel electrophoresis, without the need for costly automated electrophoresis systems (Collard and Mackill 2009). The higher primer lengths and subsequently higher annealing temperatures ensure higher reproducibility of SCoT markers, compared to RAPD markers. Since its discovery, SCoT markers have been utilized for characterizing genetic diversity in a variety of plant species (Collard and Mackill 2009; Xiong et al. 2010; Chen et al. 2010; Luo et al. 2010, 2011; Han et al. 2011; Zhang et al. 2011; Gorji et al. 2011; Deng et al. 2012; Bhattacharyya et al. 2013; Mulpuri et al. 2013; Cabo et al. 2014; Que et al. 2014).

The results of the present study, using 23 coconut accessions and 15 SCoT primers, indicated that 87.2 % of the scored fragments were polymorphic, which was relatively high compared to earlier studies utilizing dominant marker systems like RAPD (Upadhyay et al. 2004; Manimekalai and Nagarajan 2006b) and ISSR (Manimekalai and Nagarajan 2006a). Seven of the primers gave 100 % polymorphism in the 23 samples, making them comparable to polymorphism observed with SSR markers (Perera et al. 1999; Rivera et al. 1999; Merrow et al. 2003; Rajesh et al. 2008a, b, 2014). Pair wise similarity coefficient’s ranged from 0.37 to 0.91 indicating the capability of SCoT markers to detect high levels of genetic diversity among the coconut accessions analyzed. The PIC values were also much higher compared to earlier studies in coconut using RAPD markers (Manimekalai and Nagarajan 2006b).

Cluster analysis revealed grouping of coconut accessions in accordance with both their breeding habit (‘talls’ or ‘dwarfs’) and also their geographical origins. A lot of earlier studies, utilizing molecular markers, have revealed genetic distinctiveness of tall and dwarf coconut accessions, which is mainly due to the differences their allogamous/autogamous breeding behaviors (Perera et al. 1999; Rivera et al. 1999; Teulat et al. 2000). The autogamous dwarf coconut accessions display less phenotypic diversity (Zizumbo-Villarreal and Piñero 1998; Arunachalam et al. 2005) and genetic diversity in contrast to allogamous talls. Lebrun et al. (2005) have reported that regardless of their geographical origins, all dwarf coconut accessions form a genetically uniform group, under the Pacific group and the sub-group Southeast Asia. The present study with SCoT markers also corroborates the above observation. The clustering pattern based on SCoT markers also indicated that the dwarf accessions formed an uniform group within the tall accessions. Perera et al. (2000) had hypothesized the possibility that dwarf coconut accessions had evolved from a small number of tall palms because almost all the alleles present in dwarf coconut accessions were shared by tall accessions.

Tall coconut accessions have been divided into two major cultivar groups—the Pacific group and the Indo-Atlantic group (Lebrun et al. 2005). Tall coconut accessions from the same region, given their allogamous nature, might possess a comparatively similar genetic structure (Lebrun et al. 2005). Consequently, an obvious pattern of variation is expected and has been reported in many studies (Lebrun et al. 1998; Perera et al. 2000). In the present study, clustering of tall coconut accessions, according to the geographical proximities, was observed, which is in line with earlier studies using microsatellite markers (Perera et al. 2000; Rivera et al. 1999). Interestingly, the two accessions from Lakshadweep Islands (India), viz. LCT and LMMT, formed a distinct cluster. The region around the southern margins of the Indian sub-continent, comprising Lakshadweep Islands, Sri Lanka and Maldives, represent a possible center of coconut domestication (Gunn et al. 2011) and may explain the uniqueness of these two accessions.

In contrast to talls, dwarfs, being autogamous, are inclined to conserve their genetic structure regardless of the region of location. The genetic structure of dwarf accessions, therefore, is an indication of their region of origin, viz. Southeast Asia, rather than the region, which they are presently found, in spite of their growing there for an extended time (Lebrun et al. 2005). SCoT analyses revealed close relationships among the dwarf accessions from India (CGD and COD), Malaysia (MYD and MOD), Sri Lanka (SLRD and SLGD) and the Pacific Ocean region (NKOD and NLAD). Amongst dwarf accessions analyzed, one exception was NLAD, which occupied a unique position in the cluster when compared to other dwarfs when analyzed using SCoT markers. NLAD, in spite of being a dwarf, is allogamous and had showed highest diversity amongst a set of dwarf samples analyzed using microsatellites (Merrow et al. 2003) and foliar traits (Arunachalam et al. 2005).

This study indicates that SCoT markers are informative and could be used to detect polymorphism among coconut germplasm accessions. The SCoT markers could either be used individually or in combination with other molecular markers to assess genetic diversity of coconut germplasm and to obtain reliable information about population structure across coconut populations, which would aid strategies for effective collection of coconut germplasm, their conservation in genebanks and in grouping them for making further selections as well as removing duplicates.
